# Supplementary material for: Gestational diabetes in women with obesity; an analysis of clinical history and simple clinical/anthropometric measures
Source: PLoS One. 2022 Dec 30;17(12):e0279642. doi: 10.1371/journal.pone.0279642 (PMC9803279; doi:10.1371/journal.pone.0279642)
Supplement: S1 File — (DOCX) [file pone.0279642.s001.docx]

Supporting Information: Tables

S1 Table: Groups of potentially correlated maternal clinical variables

| **Groups** | **Potentially correlated variables** | | | |  |  |  |
| --- | --- | --- | --- | --- | --- | --- | --- |
| **Blood pressure** | | systolic | diastolic |  |  |  |  |
| **Skin folds** | | Suprailiac | subscapular | biceps | triceps |  |  |
| **Circumferences** | | Waist | mid-arm | neck | BMI | weight |  |
| **Body habitus** | | waist:height | waist: thigh | waist:hip | neck:thigh | BMI | weight |

S2 Table: Maternal characteristics of women included in clinical analysis, by GDM status (complete and imputed datasets)

|  | **Complete-case dataset *n*=1177** | | **Imputed dataset *n*=1303** | |
| --- | --- | --- | --- | --- |
| **Maternal factors** | **No GDM** | **GDM** | **No GDM** | **GDM** |
|  | **(*n*=873)** | **(*n*=304, 25.8%)** | **(*n*=966)** | **(*n*=337, 25.8%)** |
|  | **Mean (SD) or *n* (%)** | **Mean (SD) or *n* (%)** | **Mean or % (95% CI)** | **Mean or % (95% CI)** |
| **Age (years)** | **30.3 (5.5)** | **32 (4.9)*** | 30.3 (30.0 – 30.7) | 32.0 (31.5 – 32.5) |
| **Ethnicity** |  |  |  |  |
| African | 131 (15) | 56 (18.4) | 15.6 (13.3 – 17.9) | 19.0 (14.8 - 23.2) |
| African Caribbean | 70 (8) | 22 (7.2) | 7.7 (6.0 – 9.3) | 6.5 (3.9 - 9.2) |
| South Asian | 48 (5.5) | 19 (6.3) | 5.7 (4.2 – 7.2) | 6.2 (3.6 - 8.8) |
| European | 562 (64.4) | 182 (59.9) | 63.8 (60.7 – 66.8) | 60.5 (55.2 - 65.8) |
| Other | 62 (7.1) | 25 (8.2) | 7.2 (5.6 – 8.9) | 7.7 (4.9 -10.6) |
| **Adjusted English & Scottish IMD** |  |  |  |  |
| least deprived | 209 (23.9) | 63 (20.7) | 23.8 (21.1-26.5) | 20.5 (16.2 – 24.9) |
| Intermediate | 315 (36.1) | 100 (32.9) | 35.5 (32.5 – 38.5) | 34.3 (29.3 – 39.5) |
| most deprived | 349 (40) | 141 (46.4) | 40.7 (37.6 – 43.8) | 45.1 (39.8 – 50.4) |
| **Parity** |  |  |  |  |
| Nulliparous | 395 (45.2) | 128 (42.1) | 45.0 (41.9 – 48.2) | 42.4 (37.1 – 47.7) |
| **Previous GDM** |  |  |  |  |
| No previous | 467 (53.5) | 163 (53.6) | 53.8 (50.7 – 57.0) | 53.4 (48.1 – 58.8) |
| Previous | **11 (1.3)** | **13 (4.3)** | 1.1 (0.5 – 1.8) | 4.2 (2.0 - 6.3) |
| Nulliparous | 395 (45.2) | 128 (42.1) | 45.0 (41.9 – 48.2) | 42.4 (37.1 – 47.7) |
| **PCOS** | 80 (9.2) | 35 (11.5) | 8.9 (7.1 – 10.7) | 11.4 (8.0 – 14.8) |
| **Current Smoking** | 53 (6.1) | 24 (7.9) | 6.2 (4.7 – 7.7) | 8.3 (5.3 – 11.3) |
| **Family History** |  |  |  |  |
| T2DM | **187 (21.4)** | **96 (31.6)*** | 21.2 (18.6 – 23.8) | 31.0 (25.9 – 35.8) |
| GDM | 31 (3.6) | 15 (4.9) | 3.9 (2.6 – 5.1) | 5.4 (2.9 – 8.0) |
| IHD | 128 (14.7) | 56 (18.4) | 14.5 (12.3 -16.8) | 17.2 (13.2 – 21.3) |
| HTN | 392 (44.9) | 146 (48) | 44.6 (41.5 – 47.7) | 48.4 (43.0 – 53.7) |

**Bold** *p* value <0.05***,* bold***** <0.001 (*p* value from *t-*test or χ^2^ as appropriate). IMD Index of multiple deprivation, PCOS polycystic ovarian syndrome, T2DM type 2 diabetes, IHD ischaemic heart disease, HTN hypertension

S3 Table: Maternal clinical and anthropometric measures at time point 1 (15 - 18^+6^ weeks’) by GDM status (complete and imputed datasets)

|  | | **Complete-case dataset *n*=1177** | | | **Imputed dataset *n*=1303** | | | | | | |
| --- | --- | --- | --- | --- | --- | --- | --- | --- | --- | --- | --- |
| **Clinical Examination** | **No GDM** | | **GDM** | | | **No GDM** | | | | **GDM** | |
|  | **(*n*=873)** | | **(*n*=304)** | | | **(*n*=966)** | | | | **(*n*=337)** | |
|  | **Mean (SD) / median (IQR)** | | **Mean (SD)/ median (IQR)** | | | **Mean/Median or % (95% CI)** | | | | **Mean/ Median or % (95% CI)** | |
| Systolic BP (mmHg) | | **116.8 (10.7)** | | **120.9 (10.9)*** | | | 116.6 (116 – 117.3) | | 121.0 (119.9 – 122.2) | |  |
| Diastolic BP (mmHg) | | **71.5 (7.6)** | | **74.3 (8.1)*** | | | 71.4 (70.9 – 71.9) | | 74.4 (73.5 – 75.2) | |  |
| Weight (Kg) | | **97.1 (13.8)** | | **99.5 (16.4)** | | | 97.1 (96.2 – 98.0) | | 100.5 (98.6 – 102.4) | |  |
| Height (cm) | | **164.3 (6.7)** | | **163.5 (7.1)** | | | 164.2 (163.8 – 164.7) | | 163.7 (163.0 – 164.5) | |  |
| BMI (kg/m^2^) | | **34.7 (32.7-38.1)** | | **36 (33.1-39.7)*** | | | 34.7 (34.4 – 35.0) | | 36.2 (35.5 – 36.9) | |  |
| **Circumferences** | |  | |  | | |  | |  | |  |
| Waist (cm) | | **106.3 (9.9)** | | **110.3 (10)*** | | | 106.4 (105.7 – 107.0) | | 110.7 (109.6 – 111.9) | |  |
| Thigh (cm) | | 68.4 (6.3) | | 68.7 (7.6) | | | 68.4 (68.0 – 68.8) | | 68.9 (68.1 – 69.8) | |  |
| Wrist (mm) | | 172.2 (14.1) | | 173.5 (13.6) | | | 172.2 (171.3 – 173.1) | | 174.5 (172.9 – 176.1) | |  |
| Mid-arm (cm) | | **36.5 (3.9)** | | **37.6 (4.1)*** | | | 36.5 (36.2 – 36.7) | | 37.8 (37.3 – 38.2) | |  |
| Neck (cm) | | **36.3 (2.4)** | | **37.3 (2.6)*** | | | 36.3 (36.1 – 36.4) | | 37.4 (37.1 – 37.6) | |  |
| Hip (cm) | | 122.4 (9.7) | | 123.6 (11.4) | | | 122.4 (121.8 – 123.1) | | 124.2 (122.9 – 125.4) | |  |
| **Skin folds (mean, mm)** | |  | |  | | |  | |  | |  |
| Biceps | | **21.3 (7.5)** | | **23.5 (8.4)*** | | | 21.3 (20.8 – 21.8) | | 23.5 (22.6 – 24.4) | |  |
| Triceps | | **32.4 (8.7)** | | **34.5 (9.6)*** | | | 32.4 (31.9 – 33.0) | | 34.7 (33.6 – 35.7) | |  |
| Subscapular | | **34.4 (9.5)** | | **38.2 (10.9)*** | | | 34.4 (33.8 – 35.0) | | 38.3 (37.1 – 39.5) | |  |
| Suprailiac | | **31.4 (11)** | | **34.5 (10.8)*** | | | 31.4 (30.7 – 32.0) | | 34.7 (33.5 – 35.9) | |  |
| Sum of skinfolds | | **119.4 (25.7)** | | **130.7 (29)*** | | | 119.5 (117.8 – 121.1) | | 131.2 (128.0 – 134.3) | |  |
| **Ratios** | |  | |  | | |  | |  | |  |
| Waist:height | | **0.65 (0.06)** | | **0.68 (0.06)*** | | | 0.65 (0.64 – 0.65) | | 0.68 (0.67 – 0.68) | |  |
| Waist:thigh | | **1.56 (0.18)** | | **1.62 (0.22)*** | | | 1.56 (1.55 – 1.57) | | 1.62 (1.60 – 1.64) | |  |
| Waist:hip | | **0.87 (0.07)** | | **0.89 (0.07)*** | | | 0.87 (0.87 – 0.88) | | 0.89 (0.89 – 0.90) | |  |
| Neck:thigh | | **0.53 (0.05)** | | **0.55 (0.07)*** | | | 0.53 (0.53 – 0.54) | | 0.55 (0.54 – 0.56) | |  |
| GA at time point 1, weeks | | 17.1 (1.1) | | 17.1 (1.0) | - | | | - | | | |

**Bold** *p* value <0.05***,* bold***** *p* value <0.001 (*p* value from *t-*test or Mann Whitney as appropriate). GDM gestational diabetes, BP blood pressure, GA gestational age, SD standard deviation, IQR interquartile range

S4 Table: Maternal clinical and anthropometric measures at time point 2 (27 - 28^+6^ weeks’) by GDM status (complete and imputed datasets)

|  | | **Complete-case dataset *n*=1177** | | | | **Imputed dataset *n*=1303** | | | |
| --- | --- | --- | --- | --- | --- | --- | --- | --- | --- |
| **Clinical Examination** | | **No GDM** | | **GDM** | | **No GDM** | | **GDM** | |
|  | | **(*n*=873)** | | **(*n*=304)** | | **(*n*=966)** | | **(*n*=337)** | |
|  | | **Mean (SD) / median (IQR)** | | **Mean (SD) / median (IQR)** | | **Mean/ % (95% CI)** | | **Mean/ % (95% CI)** | |
| Systolic BP (mmHg) | | **117.5 (10.6)** | | **120.8 (10.9)*** | | 117.4 (116.7 – 118.1) | | 121.1 (119.9 - 122.3) | |
| Diastolic BP (mmHg) | | **72.4 (7.9)** | | **74.1 (7.9)** | | 72.2 (71.7 – 72.7) | | 74.2 (73.3 – 75.0) | |
| Weight (Kg) | | **101 (13.9)** | | **103.3 (16.6)** | | 101.0 (100.2 – 101.9) | | 104.3 (102.5 – 106.2) | |
| BMI (kg/m^2^) | | 36.4 (34.3 – 39.6) | | 37.7 (34.7 – 41.3) | | - | | - | |
| **Circumferences** | |  | |  | |  | |  | |
| Waist (cm) | | **112.2 (9.1)** | | **116.1 (9.4)*** | | 112.2 (111.6 – 112.8) | | 116.4(115.3 – 117.5) | |
| Thigh (cm) | | 68.8 (6.4) | | 69.2 (7.6) | | 68.8 (68.5 – 69.2) | | 69.4 (68.6 – 70.2) | |
| Wrist (mm) | | 173.3 (13.6) | | 174.1 (13.8) | | 173.4 (172.6 – 174.3) | | 175.3 (173.7 – 177.0) | |
| Mid-arm (cm) | | **36.4 (3.9)** | | **37.6 (4.1)*** | | 36.3 (36.1 – 36.6) | | 37.8 (37.4 – 38.3) | |
| Neck (cm) | | **36.3 (2.3)** | | **37.3 (2.6)*** | | 36.3 (36.2 – 36.5) | | 37.4 (37.1 – 37.7) | |
| Hip (cm) | | 124.2 (9.8) | | 125.1 (11.6) | | 124.3 (123.7 – 124.9) | | 125.6 (124.3 – 126.9) | |
| **Skin folds (mean, mm)** | |  | |  | |  | |  | |
| Biceps | | **21.4 (7.4)** | | **22.9 (7.1)** | | 21.3 (20.8 – 21.8) | | 23.1 (22.3 – 23.9) | |
| Triceps | | **32.8 (8.2)** | | **35.1 (9.1)*** | | 32.8 (32.3 – 33.3) | | 35.5 (34.5 – 36.5) | |
| Subscapular | | **35.8 (8.9)** | | **39.4 (10.3)*** | | 35.7 (35.1 – 36.2) | | 39.7 (38.5 – 40.8) | |
| Suprailiac | | **32.3 (11)** | | **35.4 (10.7)*** | | 32.3 (31.6 – 33.0) | | 35.7 (34.6 – 36.9) | |
| Sum of skinfolds | | **122.3 (25.4)** | | **132.8 (27.9)*** | | 122.1 (120.5 – 123.7) | | 134.0 (131.0- 137.1) | |
| **Ratios** | |  | |  | |  | |  | |
| Waist:height | | **0.68 (0.05)** | | **0.71 (0.06)*** | | 0.68 (0.68 – 0.69) | | 0.71 (0.71 – 0.72) | |
| Waist:thigh | | **1.64 (0.17)** | | **1.69 (0.17)*** | | 1.64 (1.63 – 1.65) | | 1.69 (1.67 – 1.71) | |
| Waist:hip | | **0.91 (0.07)** | | **0.93 (0.07)*** | | 0.91 (0.90 – 0.91) | | 0.93 (0.92- 0.94) | |
| Neck:thigh | | **0.53 (0.05)** | | **0.54 (0.06)*** | | 0.53 (0.53 – 0.53) | | 0.54 (0.54 – 0.55) | |
| GA at time point 2, weeks | | 27.8 (0.7) | | 27.8 (0.7) | | - | | - | |

**Bold** *p* value <0.05***,* bold***** *p* value <0.001 (*p* value from *t-*test). GDM gestational diabetes, BP blood pressure, GA gestational age, SD standard deviation, IQR interquartile range, BMI body mass index

S5 Table: Imputed dataset - clinical factors associated with GDM at time point 1 (15 - 18^+6^ weeks’), unadjusted and multiple regression analyses

| *n=*1303 |  | **Unadjusted analysis** | **Model 1 ^^^** | **Model 2 ^>^** |
| --- | --- | --- | --- | --- |
|  |  | OR (95% CI) | OR (95% CI) | OR (95% CI) |
| Age (years) |  | **1.06 (1.04 – 1.09)** | **1.06 (1.04 – 1.09)** | **1.06 (1.03 – 1.09)** |
| Previous GDM | No | 1 (ref) | 1 (ref) | 1 (ref) |
|  | Yes | **3.68 (1.64 – 8.25)** | **3.57 (1.49 – 8.56)** | **3.40 (1.41 – 8.19)** |
|  | Nulliparous | 0.95 (0.74 – 1.22) | 1.09 (0.83 – 1.44) | 1.18 (0.89 – 1.57) |
| 1^st^ degree T2DM | Yes | **1.66 (1.26 – 2.19)** | 1.35 (0.99 – 1.82) | 1.32 (0.97 – 1.80) |
| BMI | (kg/m^2^) | **1.06 (1.04 – 1.09)** | ~ | ~ |
| Weight | (kg) | **1.02 (1.01 – 1.02)** | ** | ** |
| Systolic BP | (per 10 mmHg) | **1.44 (1.29 – 1.62)** | **1.34 (1.19 – 1.52)** | **1.37 (1.21 – 1.55)** |
| Diastolic BP | (per 10 mmHg) | **1.64 (1.39 – 1.93)** | ~ | ~ |
| Skinfolds mean | Biceps | **1.19 (1.10 – 1.28)** | ~ | ** |
| (per 5mm) | Triceps | **1.15 (1.07 – 1.23)** | ~ | ** |
|  | Subscapular | **1.21 (1.14 – 1.29)** | **1.13 (1.06 – 1.21)** | ** |
|  | Suprailiac | **1.14 (1.08 – 1.21)** | ~ | ** |
|  | Sum of all | **1.08 (1.06 – 1.11)** | ** | **1.06 (1.03 – 1.08)** |
| Circumferences | Neck | **1.20 (1.14 – 1.26)** | **1.13 (1.07 – 1.20)** | ** |
| (cm) | Waist | **1.04 (1.03 – 1.05)** | ~ | ** |
|  | Mid arm | **1.08 (1.05 – 1.12)** | ~ | ** |
| Ratios | Waist:hip | **1.53 (1.29 – 1.82)** | ** | ~ |
| Per 0.1 increase | Waist:thigh | **1.17 (1.09 – 1.25)** | ** | ~ |
|  | Neck:thigh | **1.49 (1.20 – 1.84)** | ** | **1.59 (1.27 – 1.99)** |
|  | Waist:height | **1.97 (1.62 – 2.39)** | ** | **1.55 (1.24 – 1.93)** |

Bold p value <0.05, ^simple measures adjusted for positive simple baseline variables selected after correlation analysis plus ethnicity and parity

>ratios/summed measures adjusted for positive ratio baseline variables selected after correlation analysis plus ethnicity and parity

**not included a priori ~ not included after correlation testing

GDM gestational diabetes, OR odds ratio, CI confidence interval, BMI body mass index, BP blood pressure

S6 Table: Imputed dataset - clinical factors associated with GDM at time point 2 (27 - 28^+6^ weeks’), unadjusted and multiple regression analyses

| *n=*1303 |  | **Unadjusted analysis** | **Model 1 ^^^** | **Model 2 ^>^** |
| --- | --- | --- | --- | --- |
|  |  | OR (95% CI) | OR (95% CI) | OR (95% CI) |
| Weight | (kg) | **1.01 (1.01 – 1.02)** | ~ | ~ |
| Systolic BP | (per 10 mmHg) | **1.03 (1.02 – 1.04)** | **1.30 (1.14 – 1.48)** | **1.31 (1.52 – 1.49)** |
| Diastolic BP | (per 10 mmHg) | **1.03 (1.02 – 1.05)** | ~ | ~ |
| Skinfolds | Biceps | **1.03 (1.02 – 1.05)** | ~ | * |
| (mean, per 5mm) | Triceps | **1.04 (1.02 – 1.05)** | ~ | * |
|  | Subscapular | **1.04 (1.03 – 1.06)** | **1.15 (1.07 – 1.24)** | * |
|  | Suprailiac | **1.03 (1.02 – 1.04)** | ~ | * |
|  | Sum of all | **1.02 (1.01 – 1.02)** | * | **1.06 (1.03 – 1.09)** |
| Circumferences | Neck | **1.20 (1.14 – 1.26)** | ~ | * |
| (cm) | Waist | **1.05 (1.03 – 1.06)** | **1.03 (1.12 – 1.05)** | * |
|  | Mid arm | **1.09 (1.06 – 1.13)** | ~ | * |
|  | Hip | **1.01 (1.00 – 1.02)** | ~ | * |
| Ratios | Waist:hip | **1.68 (1.40 – 2.01)** | * | ~ |
| Per 0.1 increase | Waist:thigh | **1.20 (1.11 – 1.29)** | * | ~ |
|  | Neck:thigh | **1.48 (1.19 – 1.85)** | * | **1.60 (1.26 – 2.03)** |
|  | Waist:height | **2.29 (1.83 – 2.85)** | * | **1.78 (1.38 – 2.30)** |

**Bold** *p* value <0.05 (*p* value from logistic regression)

^simple measures adjusted for positive simple time point 2 variables selected after correlation analysis plus age, ethnicity and parity, previous GDM (gestational diabetes), family history T2DM, and intervention group

^>^ratios/summed measures adjusted for positive ratio time point 2 variables selected after correlation analysis plus age, ethnicity, parity, previous GDM, family history T2DM, and intervention group

**not included *a priori* ~ not included after correlation testing.

BP blood pressure, OR odds ratio, CI confidence interval

S7 Table: Sensitivity analysis (removal of outliers)- clinical factors associated with GDM at time point 1 (15 - 17^+6^ weeks’), unadjusted and multiple regression analyses

| *n*=835 |  | **Unadjusted analysis** | **Model 1** ^ | **Model 2 ^>^** |
| --- | --- | --- | --- | --- |
| <4SD and 15–17^+6^ weeks’ |  | OR (95% CI) | OR (95% CI) | OR (95% CI) |
| Age (years) |  | **1.06 (1.03 - 1.10)*** | **1.06 (1.03 - 1.10)*** | **1.06 (1.03 - 1.09**)* |
| Previous GDM | No | 1 (ref) | 1 (ref) | 1 (ref) |
|  | Yes | **2.97 (1.12 - 7.88)** | 2.59 (0.92 - 7.28) | 2.60 (0.91 - 7.40) |
|  | Nulliparous | 0.78 (0.57 - 1.08) | 0.86 (0.61 - 1.22) | 0.92 (0.64 - 1.30) |
| 1^st^ degree T2DM | Yes | **1.69 (1.20 - 2.38)** | 1.37 (0.94 - 2.00) | 1.33 (0.64 - 1.30) |
| BMI | (kg/m^2^) | **1.05 (1.01 - 1.09)** | ~ | ~ |
| Weight | (kg) | 1.01 (0.99 - 1.02) | ** | ** |
| Systolic BP | (per 10 mmHg) | **1.41 (1.22 - 1.63)*** | **1.34 (1.15 - 1.57)*** | **1.35 (1.16 - 1.58)*** |
| Diastolic BP | (per 10 mmHg) | **1.48 (1.21 - 1.82)*** | ~ | ~ |
| Skinfolds mean | Biceps | **1.19 (1.07 - 1.32)** | ~ | ** |
| (per 5mm) | Triceps | **1.10 (1.01 - 1.20)** | ~ | ** |
|  | Subscapular | **1.19 (1.10 - 1.29)*** | **1.12 (1.03 - 1.22)** | ** |
|  | Suprailiac | **1.13 (1.06 - 1.22)*** | ~ | ** |
|  | Sum of all | **1.08 (1.05 - 1.11)*** | ** | **1.05 (1.01 - 1.09)** |
| Circumferences | Neck | **1.17 (1.10 - 1.25)*** | ~ | ** |
| (cm) | Waist | **1.04 (1.02 - 1.06)*** | **1.11 (1.03 - 1.19)** | ** |
|  | Mid arm | **1.06 (1.02 - 1.11)** | ~ | ** |
| Ratios | Waist-to-hip | **1.82 (1.43 - 2.32)*** | ** | ~ |
| Per 0.1 increase | Waist-to-thigh | **1.22 (1.11 - 1.35)*** | ** | ~ |
|  | Neck-to-thigh | **1.64 (1.23 - 2.19)** | ** | **1.57 (1.16 - 2.12)** |
|  | Waist-to-height | **2.09 (1.59 - 2.76)*** | ** | **1.52 (1.10 - 2.10)** |

**Bold** *p* value <0.05***,* bold***** *p* value <0.001 (*p* value from logistic regression)

^simple measures adjusted for positive simple baseline variables selected after correlation analysis plus ethnicity and parity

^>^ratios/summed measures adjusted for positive ratio baseline variables. selected after correlation analysis plus ethnicity and parity

**not included *a priori* ~ not included after correlation testing.

GDM gestational diabetes, BP blood pressure, SD standard deviation, OR odds ratio, CI confidence interval

S8 Table: Sensitivity analysis (removal of outliers) - clinical factors associated with GDM at time point 2 (27 - 28^+6^ weeks’), unadjusted and multiple regression analyses

| *n=1076* | | |  | **Unadjusted analysis** | | | **Model 1 ^^^** | **Model 2 ^>^** |
| --- | --- | --- | --- | --- | --- | --- | --- | --- |
| <4SD and 27-28^+6^ weeks’ | | |  | OR (95% CI) | | | OR (95% CI) | OR (95% CI) |
| Previous GDM | No | | | 1 (ref) | 1 (ref) | | 1 (ref) |  |
|  | Yes | | | **3.11 (1.35 – 7.20)** | **2.58 (1.07 – 6.21)** | | 2.35 (0.95 – 5.77) |  |
|  | Nulliparous | | | 0.91 (0.69 – 1.21) | 1.01 (0.74 – 1.37) | | 0.92 (0.80 – 1.49) |  |
| 1^st^ degree T2DM | Yes | | | **1.96 (1.45 – 2.65)*** | **1.71 (1.24 – 2.37)** | | **1.60 (1.15 – 2.22)** |  |
| Weight | | | (kg) | 1.01 (0.99 - 1.02) | | | ~ | ~ |
| Systolic BP | | | (per 10 mmHg) | **1.29 (1.14 - 1.47)*** | | | **1.28 (1.11 - 1.48)** | **1.29 (1.12 - 1.49)*** |
| Diastolic BP | | | (per 10 mmHg) | **1.35 (1.14 - 1.61)** | | | ~ | ~ |
| Skinfolds | | | Biceps | **1.15 (1.04 - 1.26)** | | | ~ | * |
| (mean, per 5mm) | | | Triceps | **1.15 (1.06 - 1.25)** | | | ~ | * |
|  | | | Subscapular | **1.22 (1.13 - 1.31)*** | | | **1.16 (1.06 - 1.25)** | * |
|  | | | Suprailiac | **1.14 (1.07 - 1.22)** | | | ~ | * |
|  | | | Sum of all | **1.08 (1.05 - 1.11)*** | | | * | **1.06 (1.02 - 1.09)** |
| Circumferences | | | Neck | **1.18 (1.11 - 1.26)*** | | | ~ | * |
| (cm) | | | Waist | **1.04 (1.03 - 1.06)*** | | | **1.03 (1.01 - 1.04)** | * |
|  | | | Mid arm | **1.07 (1.03 - 1.11)*** | | | ~ | * |
|  | | | Hip | 1.00 (0.99 - 1.02) | | | ~ | * |
| Ratios | | | Waist-to-hip | **1.68 (1.37 - 2.07)*** | | | * | ~ |
| Per 0.1 increase | | | Waist-to-thigh | **1.20 (1.10 - 1.31)*** | | | * | ~ |
|  | | | Neck-to-thigh | **1.58 (1.23 - 2.05)*** | | | * | **1.67 (1.27 - 2.19)*** |
|  | | | Waist-to-height | **2.27 (1.75 - 2.94)*** | | | * | **1.79 (1.33 - 2.41)*** |

**Bold** *p* value <0.05***,* bold***** *p* value <0.001 (*p* value from logistic regression)

^simple measures adjusted for positive simple time point 2 variables selected after correlation analysis plus age, ethnicity and parity, previous GDM, family history t2dm, and intervention group ^>^ratios/summed measures adjusted for positive ratio time point 2 variables selected after correlation analysis plus age, ethnicity, parity, previous GDM, family history T2DM, and intervention group

**not included *a priori* ~ not included after correlation testing

GDM gestational diabetes, T2DM type 2 diabetes, BP blood pressure, SD standard deviation, OR odds ratio, CI confidence interval

S9 Table: Rate change per week between time point 1 and 2 by GDM status

|  | **No GDM** | **GDM** |  |
| --- | --- | --- | --- |
| **Rates (change per week)** | **(n=873)** | **(n=304)** | ***p* value*** |
|  | **Mean (SD) or n (%)** | **Mean (SD) or n (%)** |  |
| Weight (Kg) | 0.37 (0.26) | 0.35 (0.27) | 0.36 |
| Systolic BP (mmHg) | 0.08 (1.05) | -0.01 (1.12) | 0.18 |
| Diastolic BP (mmHg) | 0.09 (0.74) | -0.03 (0.8) | 0.015 |
| **Circumferences** |  |  |  |
| Waist (cm) | 0.56 (0.59) | 0.55 (0.47) | 0.84 |
| Thigh (cm) | 0.03 (0.41) | 0.05 (0.41) | 0.70 |
| Wrist (mm) | 0.11 (1.01) | 0.05 (1.05) | 0.38 |
| Mid-arm (cm) | -0.01 (0.19) | 0 (0.15) | 0.32 |
| Neck (cm) | 0 (0.14) | 0 (0.17) | 0.77 |
| Hip (cm) | 0.17 (0.44) | 0.14 (0.51) | 0.34 |
| **Skin folds (mean, mm)** |  |  |  |
| Biceps | 0.01 (0.64) | -0.06 (0.62) | 0.10 |
| Triceps | 0.04 (0.61) | 0.06 (0.65) | 0.61 |
| Subscapular | 0.14 (0.84) | 0.11 (0.88) | 0.71 |
| Suprailiac | 0.1 (0.89) | 0.09 (0.83) | 0.91 |
| Sum of skinfolds | 0.29 (1.99) | 0.21 (1.9) | 0.56 |
| **Ratios** |  |  |  |
| Waist:height | 0.02 (0.03) | 0.02 (0.02) | 0.96 |
| Waist:thigh | 0.05 (0.1) | 0.05 (0.11) | 0.44 |
| Waist:hip | 0.02 (0.04) | 0.02 (0.03) | 0.73 |
| Neck:thigh | 0 (0.03) | 0 (0.04) | 0.39 |

^*^*p* value from *t* test. SD standard deviation. GDM gestational diabetes

S10 Table: unadjusted and multiple regression analyses for association of GDM with rate change of diastolic BP

| **Rate, change/week** | **Unadjusted analysis** | **Adjusted analysis*** |
| --- | --- | --- |
| Diastolic BP | 0.81 (0.68 - 0.96) | 0.84 (0.70 - 1.00) |

*Adjusted for age, ethnicity, previous GDM and family history of T2DM (drops out with just age)

S11 Table: Rate change per week between time point 1 (15 - 18^+6^ weeks’) and 2 (27 - 28^+6^ weeks’) by GDM status in complete and imputed dataset

|  | **Complete-case dataset** | | **Imputed dataset** | |
| --- | --- | --- | --- | --- |
|  | **No GDM** | **GDM** | **No GDM** | **GDM** |
| **Rates (change per week)** | **(*n*=873)** | **(*n*=304)** | **(*n*=966)** | **(*n*=337)** |
|  | **Mean (SD)** | **Mean (SD)** | **Mean (95% CI)** | **Mean (95% CI)** |
| Weight (Kg) | 0.37 (0.26) | 0.35 (0.27) | 0.37 (0.35 – 0.39) | 0.36 (0.34 – 0.38) |
| Systolic BP (mmHg) | 0.08 (1.05) | -0.01 (1.12) | 0.08 (0.01 – 0.14) | -0.007 (-0.13 – 0.11) |
| Diastolic BP (mmHg) | 0.09 (0.74) | -0.03 (0.8) | 0.08 (0.04 – 0.13) | -0.03 (-0.12 – 0.05) |
| **Circumferences** |  |  |  |  |
| Waist (cm) | 0.56 (0.59) | 0.55 (0.47) | 0.55 (0.51 – 0.59) | 0.53 (0.48 – 0.58) |
| Thigh (cm) | 0.03 (0.41) | 0.05 (0.41) | 0.04 (0.01 – 0.06) | 0.04 (-0.002 – 0.08) |
| Wrist (mm) | 0.11 (1.01) | 0.05 (1.05) | 0.12 (0.05 – 0.18) | 0.07 (-0.05 – 0.20) |
| Mid-arm (cm) | -0.01 (0.19) | 0 (0.15) | -0.01 (-0.02 – 0.001) | 0.005 (-0.01 – 0.02) |
| Neck (cm) | 0 (0.14) | 0 (0.17) | 0.005 (-0.005 – 0.15) | 0.001 (-0.02 – 0.02) |
| Hip (cm) | 0.17 (0.44) | 0.14 (0.51) | 0.17 (0.14 – 0.20) | 0.14 (0.08 – 0.19) |
| **Skin folds (mean, mm)** |  |  |  |  |
| Biceps | 0.01 (0.64) | -0.06 (0.62) | 0.004 (-0.04 – 0.05) | -0.04 (-0.11 – 0.03) |
| Triceps | 0.04 (0.61) | 0.06 (0.65) | 0.04 (-0.001- 0.08) | 0.08 (0.008 – 0.15) |
| Subscapular | 0.14 (0.84) | 0.11 (0.88) | 0.13 (0.08 – 0.18) | 0.13 (0.03 – 0.22) |
| Suprailiac | 0.1 (0.89) | 0.09 (0.83) | 0.09 (0.04 – 0.15) | 0.11 (0.02 – 0.20) |
| Sum of skinfolds | 0.29 (1.99) | 0.21 (1.9) | 0.26 (0.14 – 0.39) | 0.27 (0.07 – 0.48) |
| **Ratios** |  |  |  |  |
| Waist:height | 0.02 (0.03) | 0.02 (0.02) | 0.02 (0.02 – 0.03) | 0.02 (0.02 – 0.02) |
| Waist:thigh | 0.05 (0.1) | 0.05 (0.11) | 0.05 (0.04 – 0.06) | 0.05 (0.03 – 0.06) |
| Waist:hip | 0.02 (0.04) | 0.02 (0.03) | 0.02 (0.02 – 0.03) | 0.02 (0.02 – 0.03) |
| Neck:thigh | 0 (0.03) | 0 (0.04) | -0.001 (-0.003 – 0.00) | -0.003 (-0.006 – 0.001) |

GDM gestational diabetes, SD standard deviation, CI confidence interval

S12 Table: Unadjusted and multiple regression analyses for association of GDM with rate change of diastolic BP (Imputed dataset)

| **Rate, change/week** | **Unadjusted analysis** | **Adjusted analysis*** |
| --- | --- | --- |
| Diastolic BP | 0.82 (0.69 – 0.97) | 0.85 (0.71 – 1.00) |

*Adjusted for age, ethnicity, previous GDM and family history of T2DM

S13 Table: Sensitivity analysis: rate change per week between time point 1 (15 - 17^+6^ weeks’) and 2 (27 - 28^+6^ weeks’)

by GDM status with removal of outliers

|  | **No GDM** | **GDM** |  |
| --- | --- | --- | --- |
| *n*=766 | **(*n*=563)** | **(*n*=203)** | ***p* value*** |
|  | **Mean (SD)** | **Mean (SD)** |  |
| **Rates (change per week)** |  |  |  |
| Weight (Kg) | 0.36 (0.26) | 0.37 (0.27) | 0.73 |
| Systolic BP (mmHg) | 0.08 (0.97) | -0.01 (1.07) | 0.30 |
| Diastolic BP (mmHg) | 0.09 (0.71) | -0.01 (0.77) | 0.08 |
| **Circumferences** |  |  |  |
| Waist (cm) | 0.55 (0.5) | 0.55 (0.44) | 0.95 |
| Thigh (cm) | 0.02 (0.33) | 0.05 (0.33) | 0.36 |
| Wrist (mm) | 0.12 (0.8) | 0.15 (0.86) | 0.63 |
| Mid-arm (cm) | -0.02 (0.16) | 0.01 (0.14) | 0.02 |
| Neck (cm) | 0 (0.11) | -0.01 (0.11) | 0.36 |
| Hip (cm) | 0.15 (0.37) | 0.12 (0.37) | 0.44 |
| **Skin folds (mean, mm)** |  |  |  |
| Biceps | 0.01 (0.52) | -0.07 (0.5) | 0.07 |
| Triceps | 0.02 (0.52) | 0.05 (0.56) | 0.51 |
| Subscapular | 0.08 (0.76) | 0.11 (0.88) | 0.68 |
| Suprailiac | 0.07 (0.78) | 0.03 (0.73) | 0.56 |
| Sum of skinfolds | 0.17 (1.57) | 0.12 (1.6) | 0.66 |
| **Ratios** |  |  |  |
| Waist:height | 0.02 (0.02) | 0.02 (0.02) | 0.92 |
| Waist:thigh | 0.05 (0.07) | 0.05 (0.07) | 0.52 |
| Waist:hip | 0.02 (0.03) | 0.03 (0.03) | 0.78 |
| Neck:thigh | 0 (0.02) | 0 (0.02) | 0.20 |

**p* value from *t-*test. SD standard deviation, GDM gestational diabetes

S14 Table: Gestational weight gain stratified by BMI category and GDM status

|  | **No GDM** | | | **GDM** | |
| --- | --- | --- | --- | --- | --- |
| **BMI kg/m^2^** | ***n*** | **mean GWG kg/week (SD)** | ***n*** | | **mean GWG kg/week (SD)** |
| 30 - 34.9 | 464 | 0.40 (0.25) | 118 | 0.36 (0.26) | |
| 35 - 39.9 | 277 | 0.36 (0.27) | 116 | 0.36 (0.28) | |
| ≥ 40 | 132 | 0.28 (0.26) | 70 | 0.34 (0.29) | |

*GWG gestational weight gain, SD standard deviation, GDM gestational diabetes

S15 Table: Unadjusted and adjusted analysis of the association between gestational weight gain and GDM within BMI categories

| **BMI kg/m^2^** | **Unadjusted** | **Adjusted model*** |
| --- | --- | --- |
|  | **OR (95% CI)** | **OR (95% CI)** |
| 30 - 34.9 | 0.51 (0.23 - 1.14) | 0.54 (0.23 - 1.27) |
| 35 - 39.9 | 0.93 (0.42 - 2.05) | 1.12 (0.49 - 2.59) |
| ≥ 40 | 2.35 (0.81 - 6.80) | 2.62 (0.83 - 8.23) |

*adjusted for age, previous GDM, family history T2DM, ethnicity, intervention group. OR odds ratio, CI confidence interval
